# Efficacy of different-frequency TEAS on acute pain after the total knee arthroplasty: a study protocol for a parallel group randomized trial

**DOI:** 10.1186/s13063-019-3379-3

**Published:** 2019-05-29

**Authors:** Yulin Li, Lixi Chu, Xiangming Li, Weitao Zhai, Yinghui Ma, Yong He, Yuelin Xu, Sheng Ding, Huali Gao, Jing Zhang, Bowen Ye, Jingchao Wang, Jie Yao, Chonggui Wu, Lianbo Xiao

**Affiliations:** 1 Faculty of Rehabilitation Medicine, The Shanghai University of Traditional Chinese Medicine, Shanghai, 200000 China; 2 Department of Orthopaedics, Guanghua Hospital of Integrated Traditional Chinese Medicine and Western Medicine, The Shanghai University of Traditional Chinese Medicine, Shanghai, 200000 China; 3 Guanghua Hospital of Integrated Traditional Chinese Medicine and Western Medicine, Shanghai, 200000 China; 4 Traditional Chinese Medicine and Western Medicine, Shanghai, 200000 China

## Abstract

**Background:**

Total knee arthroplasty (TKA) is an optimal option for patients with middle-to-end-stage knee osteoarthritis. However, the management of postoperative acute pain remains inefficient. Transcutaneous electrical acupoint stimulation (TEAS) is a nonpharmacological method to manage postoperative acute pain. Different frequencies of TEAS have been tested using varying parameters, but the optimal analgesic frequency remains controversial. The aim of this study was to explore the optimal analgesic frequency of TEAS for treating acute pain after the primary unilateral TKA.

**Methods/design:**

This is a double-blind, randomized controlled trial. A total of 156 patients are randomly assigned to: G1, 5 Hz TEAS; G2, 100 Hz TEAS; G3, mixed TEAS (alternative use of daily 5 Hz and 100 Hz TEAS) and G4, placebo TEAS. In the G1, G2 and G3 groups, TEAS is conducted at acupoints SP9 and GB34 of the leg that was operated on (at a wave of continuous, balanced and asymmetrical biphasic square, with a pulse width of 200 μs, and a strong but comfortable current) for 30 min prior to a 30-min rehabilitation session per day for 2 weeks. In G4 group, TEAS is delivered at a strong but comfortable current for 30 s, then the current is gradually decreased to none over the next 15 s. The primary outcomes are measured before surgery, at baseline (POD 3, before TEAS intervention), week 1 and 2 after TEAS intervention with the Numeric Pain Rating Scale and The American Knee Society Score. The secondary outcomes include: (1) Active range of motion of the knee that was operated on; (2) Surface electromyography of both quadriceps; (3) Modified 30-s sit to stand test; (4) Additional usage of analgesia; and (5) SF-36. The additional outcomes include: (1) Patients’ satisfaction rate; (2) Patient’s expectation rate; and (3) Incidence of analgesia-related side effects. To test the blinding of participants and assessors, they are asked to guess whether the subjects received active or placebo TEAS within 5 min after the latest intervention. The safety and financial cost of TEAS are assessed.

**Discussion:**

Mixed TEAS has more favorable effect on acute pain control than the placebo or 5 Hz or 100 Hz TEAS.

**Trial registration:**

ChiCTR1800016347. Date of registration was 26 May 2018. Retrospectively registered.

**Electronic supplementary material:**

The online version of this article (10.1186/s13063-019-3379-3) contains supplementary material, which is available to authorized users.

## Background

### The need for total knee arthroplasty

Osteoarthritis of the hip and knee ranks 11th among the 291 causes of disability, and 38th highest among disability-adjusted life years globally [1]. The age-standardized prevalence of global knee osteoarthritis (KOA) is 3.8%, higher in elderly women [1, 2]. The optimal treatment for middle-to-end-stage KOA is total knee arthroplasty (TKA) [1, 2] which can relieve pain, recover function and improve early stage quality of life (QoL) of KOA patients [2, 3]. The therapeutic methods for middle-to-end-stage KOA have been gradually redefined, TKA of which will remain at hand in the next few decades [4]. In 2012, more than 670,000 cases of TKA were accomplished in the USA alone [5], 94 to 97% of which were operated on for KOA [2].

### Acute pain after the TKA

Surgery is the second most common cause of persistent pain [6]. Though half of all TKA patients report postoperative pain relief [7], 44% of them still suffer continued pain at 3–4 years after the operation [6]. Inadequate post-TKA pain control can lead to adverse events [8], like knee stiffness, infection, deep venous thrombosis [5, 9], and undermine the patients’ ability to perform physical activities, like stair-climbing and heavy manual work [10]. The post-TKA pain also reduces the patients’ satisfaction with this surgery [11] in as many as 18.2% [12]. It can be said that recently developed interventional and preventive strategies have all failed to effectively control the post-TKA pain [13].

### Side effects of analgesic drugs

There are multiple ways to control post-TKA pain [14]. “The panel recommends that clinicians offer multimodal analgesia…combined with nonpharmacological interventions, for the treatment of postoperative pain…(strong recommendation, high-quality evidence)” [7]. In clinical practice, surgeons and anesthesiologists usually seek help from opioids [15]. But opioids, taken either before or after the surgery, always produce side effects that can disable physical activities and lower patient satisfaction. Patients with a history of chronic opioid use who successfully decreased their intake of opioids before TKA had substantially improved clinical outcomes that were comparable to patients who did not use opioids at all [16]. At postoperative day (POD) 1 after TKA, at least 25% of patients showed an Opioid-Related Symptom Distress Scale score > 1 for nausea, drowsiness, itchiness and fatigue [15, 17]. Long-term postoperative use of opioids may lead to opioid-induced hyperalgesia (OIH), a paradoxical response to opioids [13, 18, 19]. OIH may reduce opioid efficacy and exacerbate the pain [16].

### Advantages of transcutaneous electrical acupoint stimulation

Given the side effects of opioids, the nonpharmacological approach has been developed as an adjuvant therapy to reduce pain. Transcutaneous electrical nerve stimulation (TENS) is an economical and non-invasive technique [20, 21] with few side effects [14, 22]. TENS with electrodes placed on various acupoints is also called transcutaneous electrical acupoint stimulation (TEAS) [23]. Compared with conventional acupuncture, TEAS brings no risk of needle-induced infection and is more acceptable for those fearing needle stimulation [24]. Evidence has proven that TEAS can effectively reduce postoperative acute pain, the consumption of opioids and opioid-related side effects, and improve patient satisfaction, physical function, and promote the enhanced recovery after surgery (ERAS) [15, 25, 26].

### Objectives

To evaluate the efficacy of different frequencies of TEAS on acute pain after the TKA.

## Methods/design

A mono-center, prospective, randomized, parallel-controlled, participants-, assessor- and statistician-blinded study will be carried out to find the optimal analgesic frequency of TEAS by comparing the efficacy of different frequency on postoperative acute pain control in patients undergoing the primary unilateral TKA. This study strictly followed the principles of the Declaration of Helsinki. The study protocol had been approved by the Clinical Trial Ethics Committee of the Shanghai Guanghua Hospital of Integrated Traditional Chinese and Western Medicine (approval number: 2017-k-41-01) and registered at the Chinese Clinical Trial Registry (http://www.chictr.org.cn/listbycreater.aspx, ChiCTR1800016347). The researchers will be trained in the consistency of the symptoms and signs, the evaluation process.

### Eligibility criteria

The inclusion criteria: (1) 50–80 years old; (2) diagnosis of KOA; (3) prepared to undergo primary unilateral TKA; (4) treated with the same TKA procedures; (5) be American Society of Anesthesiologists (ASA) grades I–II; (6) treated with standard general anesthesia. The exclusion criteria: (1) hyposensitivity or impairment in the leg that was operated on; (2) having used opioids for more than 2 weeks within the preoperative 6 months; (3) having received other nonpharmacological treatments within the preoperative 2 weeks that might affect the outcomes; and (4) showing TENS contraindications, including the use of pacemakers or being allergic to nickel.

### Sample size

The sample size will be calculated using pain ratings from the previous studies [27–29]. The formula:

$$ n=\frac{2{\left({Z}_{\alpha }+{Z}_{1-\beta}\right)}^2{\sigma}^2}{\Delta ^2} $$

An effect size (△) of 2 with a standard deviation (SD) of 2.88 between low-frequency (LF) TEAS and placebo-TEAS (*n* = 33 × 4/0.85 = 156), an △ of 2.35 with a SD of 2.56 between high-frequency (HF) TEAS and placebo-TEAS (*n* = 19 × 4/0.85 = 95), an △ of 1.1 with a SD of 0.7 between mixed TEAS and placebo-TEAS (*n* = 7 × 4/0.85 = 35) [26]. Using this information, a four-group study will be established: 33 patients in each group, α = 0.05 (two-sided), β = 0.2 (80% power) [30]. Considering a possible sample loss of 15%, the final sample size will be set with 39 patients in each group.

### Recruitment strategies and enrollment

KOA inpatients who are scheduled to undergo a primary unilateral TKA between May 2018 to May 2019 will be recruited by the Department of Joint Surgery, the Shanghai Guanghua Hospital of Integrated Traditional Chinese and Western Medicine. The main recruiting mode will be propagated by a resident doctor (A). The KOA inpatients willing to participate in our study will be screened by specific researchers (B). The inpatients who meet the screening criteria will be clearly aware of their involvement in this study. The participant who needs to be transferred to the intensive care unit after a standard TKA procedure or perioperative interference will be excluded. These participants often have some complications of other organ systems, including cardiopulmonary insufficiency or damaged liver or kidney function, which may interference the baseline characteristics of the participant of this clinical trial. Those participants who transfer to the general ward will be asked to provide informed consent.

**Fig. 1:**
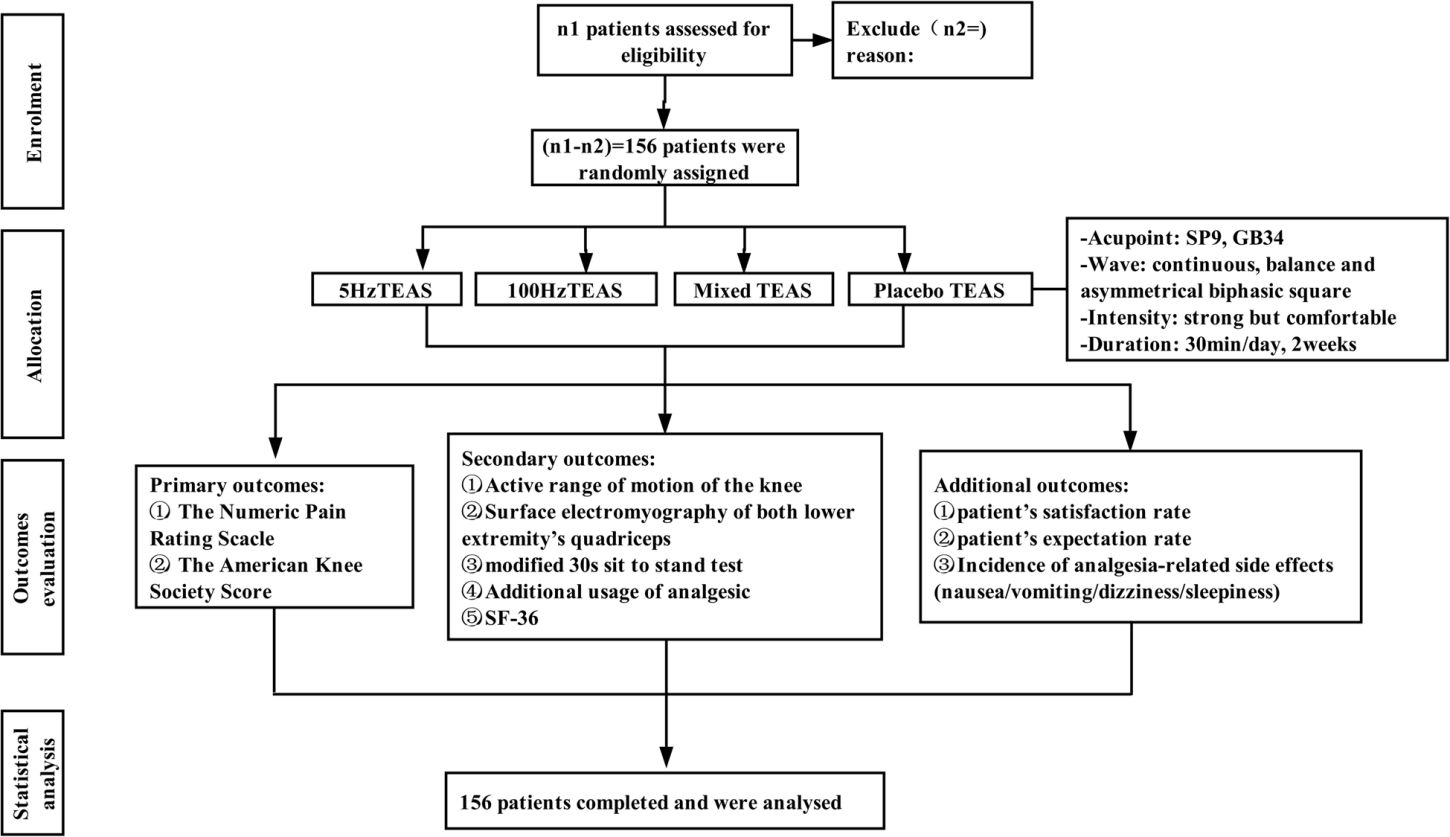
The trial flow chart

**Fig. 2:**
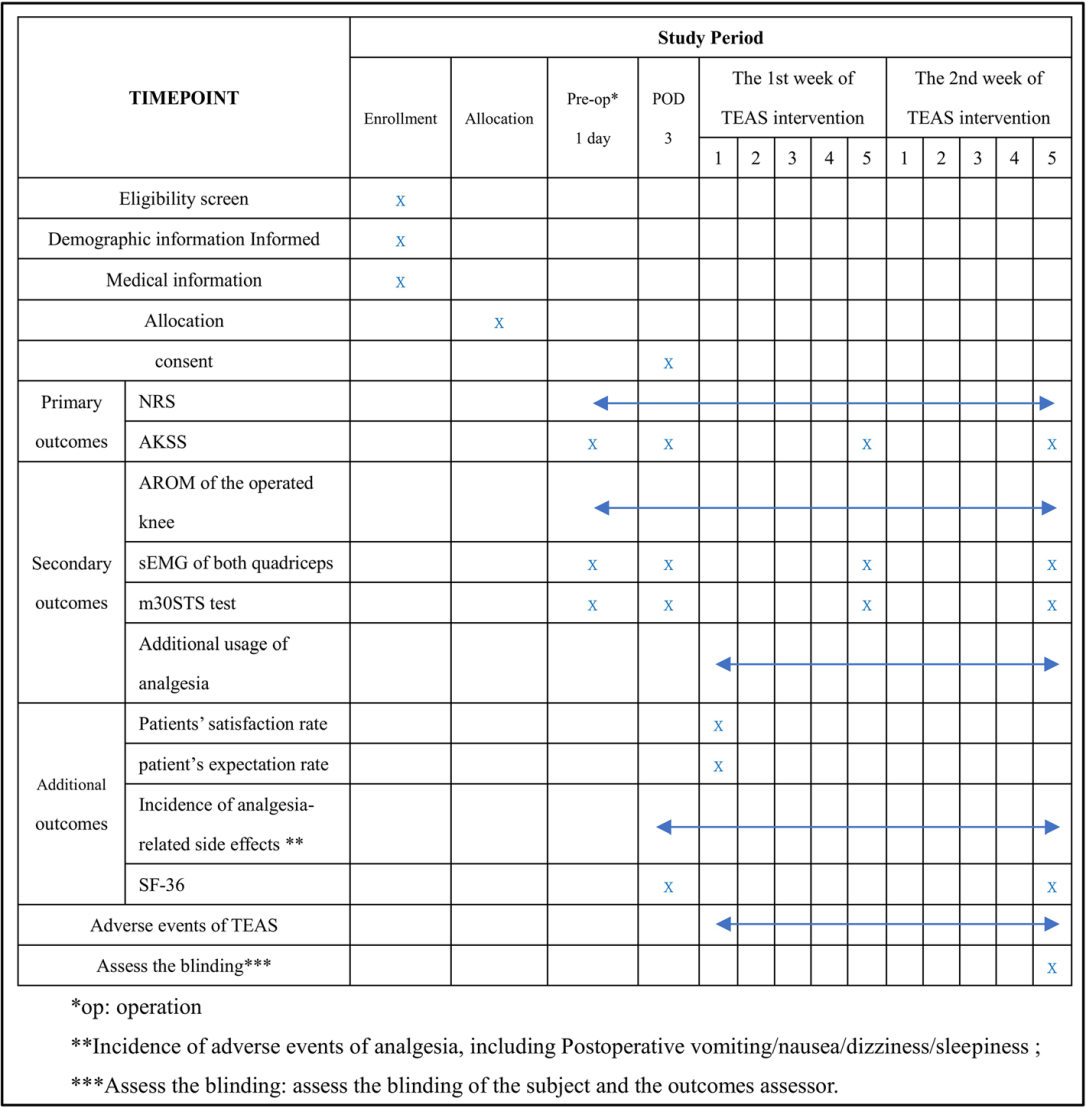
The schedule of trial enrollment, interventions and assessments

Figure 1 shows the trial flow chart for the patient screening, treatment allocation, intervention, outcomes assessment and data analysis. Figure 2 provides an overview of the study conduct, review, description and interpretation. The populated Standard Protocol Items: Recommendations for Interventional Trials (SPIRIT) Checklist is presented in Additional file 1.

### Randomisation and allocation concealment

Participants who meet the screening criteria will be randomized into four balanced groups: G1, G2, G3 and G4. The randomization sequence will be computer-generated by an independent research staff (C) with a 1:1:1:1 ratio using SPSS 21.0.

At POD 3 and before the TEAS application, three physical therapists (D, E, F) will be mainly responsible for the treatment sessions and outcomes evaluation. Initially, D will perform the basic measurements of participants in four groups and double-check whether the sensation in the leg that was operated on is normal. After D leaves the ward, E will apply a 30-min TEAS intervention and subsequently F will perform a 30-min rehabilitation session per day in four groups. One TEAS intervention and one rehabilitation constitute one treatment session. Immediately after the end of one treatment session, D will return to the ward, evaluate and record the outcomes according to the trial design. Researchers except for C and E will be blinded to the treatment allocation. Randomisation and allocation concealment in a light-tight sealed envelope until E firstly reveals the TEAS intervention according to the random number sequence at POD 3. Participants will be required not to communicate with any other researchers, participants and care providers to minimize the risk of potential bias. After the latest TEAS intervention, participants and outcome assessors will be asked to guess whether the subjects received active TEAS or placebo TEAS within 5 min to evaluate the blinding quality.

### Interventions

#### Scheme of standard analgesic use

The scheme of standard analgesics used for each participant includes two parts. First, patient-controlled analgesia (PCA) will be used from the day of surgery to the postoperative day (POD) 3, and in the meanwhile, flurbiprofen axetil (100 mg) by intravenous drip is to be administered twice a day. Second, during the POD 4 to POD 14, the participants will only intake etocoxib orally, 60 mg qid.

All participants can ask for additional analgesia if necessary. The additional consumption of analgesia (type and dosage) will be recorded daily and finally statistically analyzed.

#### TEAS interventions

TEAS will be performed using XCH-C1 equipment (ShangHai Nuo Cheng Co.). Based on the theory of Traditional Chinese Medicine, two circular electrodes (2 cm × 2 cm) will be placed around the surgical knee acupoints of SP9 (YINLINGQUAN) and GB34 (YANGLINGQUAN). SP9 is located at the hollow between the inferior edge of the medial tibial condyle and the medial edge of the tibia. GB34 is located at the lateral side of the calf in the hollow, anterior and inferior to the fibular head [31]. Before electrode attachment, E will shave and sterilize the skin with 75% ethyl alcohol. The TEAS produces a balanced, asymmetrical biphasic waveform with a pulse width of 200 μs, and a strong but comfortable current amplitude [32]. Intervention duration will be set as 30 min per day for 2 weeks [33]. Participants will be required not to change the current settings by themselves during the application of TEAS. A 30-min TEAS will be daily applied prior to a 30-min rehabilitation (including flexibility, strength, range of motion, balance and gait exercises [34]).

For the active TEAS (5 Hz/100 Hz/mixed) group, a continuous frequency of 5 Hz/100 Hz/mixed will be used. The placebo group will deliver a strong but comfortable current for 30 s which then gradually vanishes over the next 15 s. One study [35] has demonstrated that this new sham device can promote the effect of blinding through eliminating expectation bias and clarifying the true efficacy of TEAS. In this way, a sound emitted at the end of TEAS will indicate the endpoint of the TEAS treatment, which achieves the best blinding effect on participants and the outcomes assessor. Meanwhile, the participant will be told that: the TEAS works all the time during its application and they may or may not experience sensations from the TEAS unit due to different sensation thresholds of different people.

### Outcomes evaluations

Pain intensity (rest and movement) and the American Knee Society Score (AKSS) are the primary outcomes. Secondary outcomes include active range of motion (AROM) of the knee that was operated on, surface electromyography of both quadriceps, the modified 30-s sit to stand test, additional use of analgesics and the 36-item Short Form health survey (SF-36). The additional outcomes include patient satisfaction rate, patient expectation rate and the incidence of analgesia-related side effects.

The pain intensity, AROM, incidences of analgesia-related and TEAS-related side effects will be daily recorded. Evaluation of the patient satisfaction rate and patient expectation rate will be conducted after the first episode of the TEAS intervention. The SF-36 will be evaluated at two time points: before the TEAS intervention, and 2 weeks after the TEAS intervention. The assessment time points of the remaining outcomes are before the surgery, after the surgery before the TEAS intervention(at POD 3), and 1 week and 2 weeks after the TEAS intervention.

#### Demographic and medical variables

Preoperative demographic information includes sex, age, height, weight, duration of KOA, type and duration of complication, marital status, occupation, racial background, education level, preoperative medication and history of major operations.

#### Pain intensity (resting and movement)

Pain intensity is usually measured based on an 11-point Numerical Rating Scale (0–10 NRS) [36, 37]. The NRS is a horizontal line marked with the numbers 0–10 at equal intervals where 0 indicates “no pain” and 10 indicates “worst pain imaginable.” The NRS is the preferred intensity scale rather than Verbal Descriptor Scale, and Visual Analog Scale with a lower error rate and standard validity [38]. Most importantly, attributes of the NRS are not related to patient age.

Pain intensity evaluation will be carried out at rest and with movement. Prior to other procedures, the pain intensity at rest is evaluated in a position where the participant lies comfortably and still. The pain intensity at the maximum active flexion of the knee that was operated on is recorded as the pain intensity with movement.

#### The American Knee Society Score (AKSS)

The AKSS is commonly used for outcomes evaluation after the TKA globally [39], including knee assessment (0–100) and functional assessment (0–100), a total of 200 points [40]. The knee assessment consist of three components: pain (0–50), range of motion (0–25) and stability (0–25). Deduction is determined by the presence and severity of flexion contracture (0–15), extension lag (EL, 0–15) and malalignment (0–20). Function is evaluated via walking (0–50) and climbing stairs (0–50) with deductions for walking aid (0–20). For each part, a negative score is possible and should be transformed to zero.

The knee stability evaluation consists of two parts: the anterior-posterior stability test and the lateral stability test. The procedure of the anterior-posterior stability test is the same as that of the anterior drawer test of the knee. During the lateral stability test, the surgical knee is flexed by 30°. The participant will be required to stand when measuring the lower extremity alignment, and the acute angle between two lines (anterior superior iliac spine to the midpoint of the knee, the midpoint of the knee to the midpoint of the ankle) will be recorded. Valgus of 5–10° is considered to be the normal alignment and three scores are deducted for each degree of malalignment outside this range [41]. For measuring EL, a towel roll will be placed under the heel of the knee that was operated on. The participant will be required to extend their knee as much as possible, and keep the knee as straight as possible to raise the lower extremity off the towel roll. The physical therapist keeps the goniometer in alignment to measure the degrees from the straight position. The number of degrees is subtracted from the knee extension AROM to obtain the degrees of EL [42].

#### AROM measurement of the operated knee

In order to reduce the measurement error to the greatest extent, the knee AROM will be measured using a standard goniometer by the same physical therapist [43]. Bone marks of the great trochanter, the lateral epicondyle of the femur and the lateral malleolus will be firstly identified. The axis of the goniometer is positioned over the center of the lateral epicondyle of the femur. The distal arm is aligned with the fibula using the lateral malleolus and the proximal fixed arm is aligned with the femur using the greater trochanter. During the measurement, the participants will be asked to flex or extend the knee that was operated on to the maximal extent of pain that they could tolerate. Supine position is adopted for measuring the knee AROM. During the measurement of the AROM of knee flexion, participants will be required to slide the heel towards the buttocks by themselves to the greatest extent possible. When measuring the measurement of the operated knee (AROM) of knee extension, participants will be asked to extend the knee where a towel roll is placed under the heel as straight as possible. A positive value indicates a flexion position when the initial position of the knee is at the maximum extension position. AROM will be measured in triplicate for calculating the average AROM value.

#### Modified 30-s sit to stand (m30STS) test

The m30STS test will be conducted to assess the physical performance of the elderly, ensuring that people with lower body functions can also complete the test [44].

Before the m30STS test, the evaluator should first clearly introduce it to the participants. A trial is needed prior to the real test. The participant will be required to sit in the middle of the standard armchair (17 in. in height and 18 in. in width), back straight, feet shoulder-width apart, and placed on the floor at an angle slightly back from the knee. Instructions to the participate are: “When I say ‘1, 2, 3 start’, you stand up and then sit down again with both your lower limbs together as much as possible. Try your best to stand up and sit down as many times as possible until the end of 30 s. You can use your hands to help you stand up or sit down if necessary, and I will stand by your side to protect you.” The evaluator will record the number of STS repetitions.

#### Surface electromyographic (sEMG) of both quadriceps

Surface EMG will be measured simultaneously with the m30STS test. The sEMG data will be collected using a TeleMyo 2400 wireless sEMG tester (Noraxon, USA) for three major muscles of both leg: vastus medialis (VM), rectus femoris (RF) and vastus lateralis (VL), with six tunnels in total, and 1500 Hz of the sampling frequency. The electrode is a disposable ECG electrode (Ag/Cl, T-800).

Participants will be requested to wear loose pants to expose the thigh prior to sEMG. For securing the quality of the sEMG signal, the evaluator should shave the skin, sterilized with 75% alcohol and allow it to evaporate before attachment of the electrodes. Bipolar electrodes must be placed parallel to the estimated longitudinal axis of the muscle fibers below, including RF (the midpoint between the anterior superior iliac spine and the upper margin of the patella), VM (the evaluator places a towel roll under the non-surgical knee’s popliteal fossa and asks the participant to extend the knee to press the towel roll forcefully, then the bulging area slightly proximal and medial to the patella is the muscle belly of VM. Since the quadriceps of the knee that was operated on after surgery are so weak making finding the muscle belly difficult, the same area of the non-surgical knee symmetrical to the operated one, therefore, will be selected to perform the test) and VL (the midpoint between the greater trochanter and the inferior margin of the patella). The center-to-center electrode distance is 2 cm. The reference electrode is placed over the fibula head. For fastening the electrodes, the evaluator will use athletic tape for adding security without additional pressure on the top of the electrodes. Finally, fastening the wires against the body will prevent the electrodes from being pulled off, but the wires will not be placed in a way that alters the movement patterns of the participants.

A pressure transducer, instead of a high-speed camera, will be used to objectively identify the time point. The seventh tunnel (the type of tunnel is “test TTL”) will be connected to the FSR400 force-sensitive resistance transducer (the sensitivity ranges from 0 to 10 kg) to measure the change of the buttocks’ pressure during the m30STS test. The pressure signals can be transported simultaneously to the computer by a mainboard (Arduino UNO). The start time point of standing up (t1) and the end time point of sitting down (t2) will be precisely identified once the pressure value is obtained by the A0 port (range 0–1023) of mainboard exceeds 200. Data exported will be analyzed by Matlab programming. Firstly, the sEMG signals exported in the middle three STS repetitions will be denoised using a 10–400-Hz bandpass filter. The duration (t2–t1) of one STS test will be standardized by unifying interpolation of 10,000 data points. Root mean square (RMS), peak value and integrated EMG (iEMG) will be finally calculated after duration standardization. Each index will be tested three times to record the average value for statistic analysis. An engineer blinded to treatment allocation will conduct a sEMG analysis.

#### SF-36

Assessment of musculoskeletal disease consists of two parts: the expected outcome of a particular treatment and the effect of a particular treatment on the function, QoL and satisfaction of participants. The combination of general instruments and specific questionnaires may be the best way to evaluate the surgical outcomes, since misinterpretations and conclusions with limited reliability can be efficiently avoided [45]. In this study, the specific AKSS questionnaire and the generic instrument of the SF-36 will be utilized.

The SF-36 consists of eight scaled scores covering 36 items: vitality, physical functioning, bodily pain, general health perceptions, physical role functioning, emotional role functioning, social role functioning and mental health. Each scale independently produces the score from 0 to 100 and the lower score indicates a worse disability.

### The additional outcomes

Both physical factors and psychological factors contribute to dissatisfaction [46]. Hence, the patient satisfaction rate or expectation rate will be used to control the psychological factors. Participants will be asked to rank their satisfaction with a 5-point scale (1 = very unsatisfactory; 2 = unsatisfactory; 3 = neutral; 4 = satisfactory; 5 = very satisfactory), and the frequency of the “very satisfactory” determines the satisfactory ratio [47]. Patient’s expectation rating is ranked with 3-point scale (1 = positive; 2 = neutral; 3 = negative). The incidence of side effects of analgesia (nausea/vomiting/dizziness/sleepiness) and the TEAS (local irritation, redness, contact dermatitis, or muscle ache) will be recorded daily.

### Statistical analysis

All data will be analyzed using the statistical package for IBM SPSS Statistics V21. The intention-to-treat (ITT) and per-protocol sets (PP) will be analyzed. Continuous data are presented as mean ± standard deviation (SD), and categorical variables are described as percentages. Baseline data will be collected and analyzed. Continuous data with normal distribution and homogeneity variance will be analyzed using analysis of variance (ANOVA). Otherwise, the Kruskal-Wallis *H* test will be performed.

Statistical analysis includes the following four aspects. Firstly, the repeated measurement data will be analyzed using the mixed-effects model or generalized estimating equation (GEE) or generalized linear mixed models (GLMMs). Secondly, the correlation between different outcomes will be analyzed using the multiple linear regression or logistic regression. In this study, NRS, sEMG or AROM are independent variables, whereas the AKSS or m30STS test are dependent variables. Psychological factors serve as covariance. Thirdly, the safety analysis of TEAS will be compared between groups using *χ*^2^ test or Fisher’s exact test. Fourthly, health economic evaluation (cost-effectiveness analysis) will be performed. A *P* value < 0.05 (two-side) is considered statistically significant.

## Discussion

The management of postoperative acute pain remains a challenge. In clinical practice, opioids are essential in the management of postoperative acute pain although multiple opioid-sparing approaches have been developed [48]. However, opioid-related side effects markedly limit therapeutic efficacy and patient satisfaction. Thus, nonpharmacological therapies adjunct to the multimodal analgesic regimen are urgently required [49]. TEAS, a nonpharmacological and noninvasive treatment, is commonly used for the management of postoperative acute pain with fewer adverse effects.

The frequency of TEAS varies from low (LF < 10 Hz) to high frequency (HF > 50 Hz) [21]. The gate control theory and endogenous opioids mechanism are involved in TEAS [20]. The gate control theory explains the analgesic effect of HF stimulation. Compared to LF, HF is more effective in selectively activating large-diameter Aβ afferent sensory fibers and δ opioid receptors in the spinal cord [50] without simultaneously exciting small-diameter C fibers to reduce the transmission of noxious stimuli. HF TEAS exerts a segmental analgesic effect on localizing the specific dermatome with a rapid onset and offset. LF stimulation can be explained by the endogenous-opioid mechanism. LF activates endogenous opioids through stimulating small-diameter Aδ afferent sensory fibers and μ opioid receptors (MORs) [50]. Subsequently, endogenous opiates are released to provide the pain control effect. The analgesic effect of LF is extra-segmental, delayed but lasting. However, repeated electrical stimulation can also lead to opioid tolerance and cross-tolerance equivalent to repeated administration of μ or δ opioid receptor agonists [51]. The separated application of TEAS at 5 Hz and 100 Hz at alternative days may delay the tolerance effect [52, 53].

The frequency of TEAS is a key parameter for the effective management of postoperative acute pain control, while the optimal frequency remains controversial.

In vivo studies [50, 54] have suggested that both LF and HF electrical stimulation provide analgesic effect and strengthen the endogenous analgesic system in experimental animals without side effects. But Xiang et al. [55] revealed that 2-Hz TEAS but not 100-Hz TEAS induced an upregulation of MOR binding potential in numerous pain-related brain areas, and such effects even last until the end of the 2-Hz TEAS intervention in some brain areas. Yu et al. [24] yielded identical results in a rat model that shows that LF TEAS efficiently alleviated the neuropathic pain by regulating the positive expression of MORs in the dorsal root ganglion. In a clinical research, conducted by Huang et al. [26], mixed TEAS considerably reduced the demand for opioids and relieved postoperative acute pain compared to 2 Hz and 100 Hz TEAS alone. One hundred Hz TEAS can decrease the incidence of postoperative nausea and vomiting in patients undergoing video-assisted thoracic surgery. Tokuda et al. [56] also found that compared to a placebo group and a control group, the modulated-frequency electrical stimulation group had a lower pain score at rest and with movement, and better pulmonary function. However, they did not compare curative effect between modulated-frequency and LF or HF electrical stimulation. Desantana et al. [29] and Pitangui et al. [57] demonstrated that both HF and LF stimulation considerably reduced the intensity of postoperative pain compared to the placebo group. The analgesic effects between the LF and HF group was not compared. However, Oliveira et al. [56] concluded that different frequencies (0 Hz, 7 Hz, 100 Hz and 255 Hz) of electrical stimulation applied to C6–C8 did not show considerable change in the pressure-induced pain threshold or cold-stimulation-induced discomfort in healthy adults. A Cochrane review reported by Walsh et al. [58] in 2011 demonstrated that it is unable to make any definitive conclusions about the effectiveness of electrical stimulation as an isolated treatment for acute pain in adults. Johnson et al. [59] updated this Cochrane review in 2015 by adding another seven studies. They provided preliminary evidence that active electrical stimulation could decrease the acute pain in adults compared to sham electrical stimulation, but this conclusion may come with a high risk of bias because of inappropriate research design. Consistent with previous research, Bjordal [60] found that electrical stimulation notably decreased the demand for postoperative analgesics. However, a system review oppositely demonstrated that HF stimulation was effective in pain control and whether LF stimulation was better than standard care after TKA remains controversial [61]. Another system review obtained an opposite conclusion that the frequency of electrical stimulation had no effect on hypoalgesia [62].

We speculated that inappropriate research design and TEAS parameter selection may explain these controversial conclusions among previous studies. Lack of description of treatment allocation, insufficient blindness control or inappropriate sham method may significantly discount research credibility [59, 63–65]. Besides, inappropriate selection of TEAS parameters may lead to poor quality of the TEAS intervention, thus affecting the efficacy of postoperative acute pain management [61, 65]. Selection of intervention parameters of TEAS is decisive in nonpharmacological therapies [65].

In this study, the research design and selection of TEAS parameters will be modified. The sample size, admission time balance and blinding effect are strictly controlled [35]. In the previous studies aiming to find the optimal frequency, 2 Hz, 4 Hz or 5 Hz were frequently used for investigating the analgesic effect of low frequency, and 80 Hz or 100 Hz were frequently used for investigating the analgesic effect of high frequency [26, 29, 51, 55, 66]. The purpose of this clinical trial is to evaluate the different analgesic effects of low frequency, high frequency and mixed frequency on acute pain after the TKA, rather than evaluating different analgesic effects between different frequency parameter inside the high frequency or low frequency. So we will choose 5 Hz as a representative of low frequency, and 100 Hz as a representative of high frequency. A strong but comfortable current amplitude reported by the participant will be selected to achieve the best analgesic effects [32]. An intervention duration of 30 to 40 min was demanded for the complete expression of pain control effect [33]. The only variable factor of this study is the frequency of TEAS, so the non-research factors which may interfere with the outcomes are well controlled. Thus, stimulation parameters including a strong but comfortable current amplitude, 30-min intervention duration and 200-μs pulse width are consistent in all groups.

Therefore, we hypothesize that the mixed use of TEAS at different frequencies can improve postoperative acute pain, the muscle performance and knee function.

## Additional file

Additional file 1: SPIRIT 2013 Checklist. (DOC 140 kb)

## Abbreviations

AKSS: The American Knee Society Knee Score
ANOVA: Analysis of variance
AROM: Active range of motion
ASA: The American Society of Anesthesiologists
ERAS: The Enhanced Recovery After Surgery
HF: High frequency
iEMG: Integrated electromyography
ITT: Intention-to-treat analysis
KOA: Knee osteoarthritis
LF: Low frequency
m30STS: Modified 30-s sit to stand test
MOR: μ opioid receptor
NRS: Numeric rating scale
OA: Osteoarthritis
OIH: Opioid-induced hyperalgesia
ORSDS: Opioid-Related Symptom Distress Scale
POD: Postoperative day
PP: Per-protocol sets analysis
QoL: Quality of life
RF: Rectus femoris
RMS: Root mean square
SD: Standard deviation
sEMG: Surface electromyography
TEAS: Transcutaneous electrical acupoint stimulation
TENS: Transcutaneous electrical nerve stimulation
TKA: Total knee arthroplasty
VL: Vastus lateralis
VM: Vastus medialis
